# Supplementary material for: Abscisic Acid Synthesis and Signaling during the Ripening of Raspberry (Rubus idaeus ‘Heritage’) Fruit
Source: Plants (Basel). 2023 May 5;12(9):1882. doi: 10.3390/plants12091882 (PMC10180958; doi:10.3390/plants12091882)
Supplement: Supplementary file 1 [file plants-12-01882-s001.zip › Table S1.pdf]

Table S1. *RiNCED* gene family identified in *Rubus idaeus* genome.

**Gene name**	**Gene accession**	**Scaffold**	**Coordinates**	**Strand**	**Gene (bp)**	**CDS (bp)**	Protein (aa)
RiNCED1	Rr029649.t1	L2SC0000012	93872 - 91414	-	2458	1845	614
RiNCED2	Rr040293.t1	XFSC0000016	290470 – 287800	-	2670	1815	604
RiNCED3	Rr017595.t1	SC0000182	61755 - 59750	-	2005	1842	613
