# Supplementary material for: Abscisic Acid Synthesis and Signaling during the Ripening of Raspberry (Rubus idaeus ‘Heritage’) Fruit
Source: Plants (Basel). 2023 May 5;12(9):1882. doi: 10.3390/plants12091882 (PMC10180958; doi:10.3390/plants12091882)
Supplement: Supplementary file 1 [file plants-12-01882-s001.zip › Table S2.pdf]

Table S2. *RiPYL* gene family identified in *Rubus idaeus* genome.

Gene name	Gene accession	Scaffold	Coordinates	Strand	Gene (bp)	CDS (bp)	Protein (aa)
RiPYL1	Rr06488.t1	L2SC0000042	121083 - 122026	+	944	633	210
RiPYL2	Rr01684.t1	L3SC0000019	807468 - 806332	-	1137	573	190
RiPYL3	Rr026998.t1	XPSC0002114	139134 - 138300	-	950	651	216
RiPYL4	Rr05635.t1	L2SC0000037	359615 - 361019	+	1405	657	218
RiPYL5	Rr019990.t1	XFSC0000017	335895 - 336644	+	750	525	174
RiPYL6	Rr019991.t1	XFSC0000017	339324 - 338476	-	849	483	160
RiPYL7	Rr030173.t1	L3SC0000013	1287089 - 1289189	+	2101	579	192
RiPYL8	-	SC0000043	1170471 - 1173443	+	2973	555	184
RiPYL9	-	SC0000030	583417 - 582475	-	943	642	213
