# Supplementary material for: Abscisic Acid Synthesis and Signaling during the Ripening of Raspberry (Rubus idaeus ‘Heritage’) Fruit
Source: Plants (Basel). 2023 May 5;12(9):1882. doi: 10.3390/plants12091882 (PMC10180958; doi:10.3390/plants12091882)
Supplement: Supplementary file 1 [file plants-12-01882-s001.zip › Table S3.pdf]

Table S3. Information about NCED protein sequences used for phylogenetic analysis.

Species	Protein name	Accession NCBI
Arabidopsis thaliana	AtNCED2	NP_193569.1
Arabidopsis thaliana	AtNCED3	NP_188062.1
Arabidopsis thaliana	AtNCED5	NP_174302.1
Arabidopsis thaliana	AtNCED6	NP_189064.1
Arabidopsis thaliana	AtNCED9	NP_177960.1
Arabidopsis lyrata	AlNCED2	XP_002870052.2
Arabidopsis lyrata	AlNCED3	XP_002885045.1
Arabidopsis lyrata	AlNCED4	XP_002869997.1
Arabidopsis lyrata	AlNCED6	XP_002885610.1
Arabidopsis lyrata	AlNCED9	XP_002862994.1
Brassica napus	BnNCED3	NP_001302902.1
Capsella rubella	CarNCED2	XP_006282855.1
Camelina sativa	CasNCED6	ADE80897.1
Chorispora bungeana	CbNCED1	AAS68632.2
Citrus clementina	CcNCED3_1	XP_006442236.1
Citrus clementina	CcNCED5	XP_006449708.1
Citrus clementina	CcNCED6	XP_006420602.2
Citrus sinensis	CisNCED6	XP_006489810.2
Cryptomeria japonica	CjNCED	BAF31898.1
Caragana korshinskii	CkNCED1	ACU86971.1
Chrysanthemum x morifolium	CmNCED3a	BAF36657.2
Coffea canephora	CocNCED3	ABA43901.1
Cuscuta reflexa	CrNCED1	AAY82458.1

Cuscuta reflexa	CrNCED2	AAY82459.1
Capsella rubella	CrNCED3	XP_006297265.1
Capsella rubella	CrNCED5	XP_006307061.1
Capsella rubella	CrNCED6	XP_006300091.1
Capsella rubella	CrNCED9	XP_006302015.2
Citrus sinensis	CsNCED3	AER70359.1
Cucumis sativus	CsNCED6	XP_004150345.1
Daucus carota	DcNCED1	NP_001316102.1
Daucus carota	DcNCED2	NP_001316098.1
Daucus carota	DcNCED3	NP_001316101.1
Dianthus caryophyllus	DicNCED1	BAN15738.1
Eutrema salsugineum	EsNCED3	XP_006407071.1
Eutrema salsugineum	EsNCED5	XP_006415513.1
Eutrema salsugineum	EsNCED6	XP_006418817.2
Eutrema salsugineum	EsNCED9	XP_006389988.2
Fragaria x ananassa	FaNCED1	ADU85829.1
Gossypium hirsutum	GhNCED1	ADG03462.1
Gentiana lutea	GlNCED1	AAS47838.1
Glycine max	GmNCED1	NP_001241616.2
Ipomoea nil	InNCED1	XP_019159612.1
Lactuca serriola	LasNCED4	AFA36207.1
Lycium barbarum	LbNCED2	AFO85470.1
Lilium formosanum	LfNCED3	ACX33872.1
Lactuca sativa	LsNCED1	XP_023732975.1
Lactuca sativa	LsNCED2	BAE72091.1

Lactuca sativa	LsNCED3	BAE72092.1
Lactuca sativa	LsNCED4	BAE72093.1
Malus domestica	MdNCED1	AGQ03804.1
Narcissus tazetta	NtNCED3	AGT28471.1
Nicotiana tabacum	NitNCED3	NP_001313039.1
Pisum sativum	PsNCED2	BAC10550.1
Populus trichocarpa	PtNCED2	XP_002316871.1
Populus trichocarpa	PtNCED3	XP_006370181.1
Populus trichocarpa	PtNCED6	XP_002304701.2
Ricinus communis	RcNCED3	EEF32866.1
Ricinus communis	RcNCED5	EEF42638.1
Ricinus communis	RcNCED6	EEF44272.1
Ricinus communis	RcNCED9	EEF32866.1
Raphanus sativus	RsNCED1	BAF42336.1
Scutellaria baicalensis	SnNCED2	AGN03861.1
Solanum tuberosum	StNCED6	XP_006349526.1
Solanum lycopersicum	SlNCED6	XP_010321597.1
Solanum tuberosum	StNCED1	NP_001275103.1
Solanum tuberosum	StNCED2	NP_001274963.1
Vitis vinifera	VvNCED1	AAR11193.1
Vitis vinifera	VvNCED2	NP_001268200.1
Vitis vinifera	VvNCED6	XP_002283185.1
Physcomitrium patens	PpNCED4	XP_024365588.1
Physcomitrium patens	PpNCED3	XP_024397835.1
Arachis hypogaea	AhNCED1	CAE00459.2
