# Supplementary material for: Abscisic Acid Synthesis and Signaling during the Ripening of Raspberry (Rubus idaeus ‘Heritage’) Fruit
Source: Plants (Basel). 2023 May 5;12(9):1882. doi: 10.3390/plants12091882 (PMC10180958; doi:10.3390/plants12091882)
Supplement: Supplementary file 1 [file plants-12-01882-s001.zip › Table S4.pdf]

Table S4. Information about NCED protein sequences used for phylogenetic analysis. PYL protein sequences used in phylogenetic analysis. The sequences of PYLs from *Arabidopsis thaliana, Fragaria vesca, Malus domestica, Oryza sativa, Setaria italica, Vitis vinifera, Zea may*s, *Ricinus comunis, Physcomitrium patens*, and *Theobroma cacao* are available in National Center for Biotechnology Information (https://www.ncbi.nlm.nih.gov/). *Triticum aestivum, Hordeum vulgare, Sorghum bicolor, Brachypodium distachyon* genomes are available in the Ensembl plants https://plants.ensembl.org/index.html. *Nicotiana tabacum* sequences are available in Bai et al., 2019.

Specie	Protein name	Accession ID
Arabidopsis thaliana	AtPYL1	NP_001331305.1
Arabidopsis thaliana	AtPYL10	NP_194521.2
Arabidopsis thaliana	AtPYL11	NP_199398.1
Arabidopsis thaliana	AtPYL12	NP_199399.1
Arabidopsis thaliana	AtPYL13	NP_193597.1
Arabidopsis thaliana	AtPYL2	NP_180174.1
Arabidopsis thaliana	AtPYL3	NP_177443.1
Arabidopsis thaliana	AtPYL4	NP_565887.1
Arabidopsis thaliana	AtPYL5	NP_196163.1
Arabidopsis thaliana	AtPYL6	NP_565928.1
Arabidopsis thaliana	AtPYL7	NP_567208.1
Arabidopsis thaliana	AtPYL8	NP_200128.1
Arabidopsis thaliana	AtPYL9	NP_563626.1
Fragaria vesca	FvPYL13a	XP_004293952.1
Fragaria vesca	FvPYL13b	XP_004293951.1
Fragaria vesca	FvPYL13c	XP_011460608.1
Fragaria vesca	FvPYL2	XP_004291031.1
Fragaria vesca	FvPYL3	XP_004300241.1
Fragaria vesca	FvPYL4	XP_004302617.1
Fragaria vesca	FvPYL6	XP_004308958.1
Fragaria vesca	FvPYL7	XP_004306686.1
Fragaria vesca	FvPYL8a	XP_011470277.1
Fragaria vesca	FvPYL8b	XP_004308004.1

Fragaria vesca	FvPYL8c	XP_011470277.1
Malus domestica	MdPYL1	XP_008350612.1
Malus domestica	MdPYL10	XP_008377517.1
Malus domestica	MdPYL11	XP_028945528.1
Malus domestica	MdPYL12	XP_008351952.1
Malus domestica	MdPYL13	XP_008341960.1
Malus domestica	MdPYL2	XP_008343633.2
Malus domestica	MdPYL3	XP_008380616.1
Malus domestica	MdPYL4	XP_008370174.2
Malus domestica	MdPYL5	XP_008371472.1
Malus domestica	MdPYL6	XP_008373570.1
Malus domestica	MdPYL7	XP_008376112.1
Malus domestica	MdPYL8	XP_008369296.1
Malus domestica	MdPYL9	XP_008376697.2
Oryza sativa Japonica	OsPYL1	XP_015612857.1
Oryza sativa Japonica	OsPYL10	XP_015627042.1
Oryza sativa Japonica	OsPYL11	XP_015627042.1
Oryza sativa Japonica	OsPYL12	XP_025878812.1
Oryza sativa Japonica	OsPYL13	BAD46118.1
Oryza sativa Japonica	OsPYL2	XP_015641755.1
Oryza sativa Japonica	OsPYL3	XP_015624786.1
Oryza sativa Japonica	OsPYL4	XP_015643625.1
Oryza sativa Japonica	OsPYL5	XP_015638073.1
Oryza sativa Japonica	OsPYL6	XP_015628032.1
Oryza sativa Japonica	OsPYL7	XP_015641993.1
Oryza sativa Japonica	OsPYL8	XP_015641857.1
Oryza sativa Japonica	OsPYL9	XP_015642160.1
Setaria italica	SiPYL1	XP_004983564.1
Setaria italica	SiPYL2	XP_004951427.1
Setaria italica	SiPYL3	XP_004966082.1
Setaria italica	SiPYL4	XP_004961771.1

Setaria italica	SiPYL5	XP_004970455.1
Setaria italica	SiPYL6	XP_012700774.1
Setaria italica	SiPYL7	XP_004984885.1
Setaria italica	SiPYL8	XP_004951286.1
Setaria italica	SiPYL9	XP_004960624.1
Vitis vinifera	VvPYL11	XP_002272779.1
Vitis vinifera	VvPYL2	XP_010648333.1
Vitis vinifera	VvPYL4	XP_002277562.1
Vitis vinifera	VvPYL6	XP_002272779.1
Vitis vinifera	VvPYL8	XP_002270037.3
Vitis vinifera	VvPYL9	XP_002281200.1
Zea mays	ZmPYL1	NP_001343946.1
Zea mays	ZmPYL10	NP_001336882.1
Zea mays	ZmPYL11	NP_001140789.1
Zea mays	ZmPYL12	XP_035821091.1
Zea mays	ZmPYL13	PWZ11971.1
Zea mays	ZmPYL2	NP_001150686.2
Zea mays	ZmPYL3	NP_001170038.1
Zea mays	ZmPYL4	NP_001136477.1
Zea mays	ZmPYL5	NP_001148260.1
Zea mays	ZmPYL6	NP_001306656.1
Zea mays	ZmPYL7	NP_001169534.1
Zea mays	ZmPYL8	NP_001334791.1
Zea mays	ZmPYL9	NP_001140969.1
Triticum aestivum	TaPYL1A	TraesCS1A01G191700
Triticum aestivum	TaPYL1B	TraesCS1B01G206600
Triticum aestivum	TaPYL1C	TraesCS1D01G195300
Triticum aestivum	TaPYL2A	TraesCS3A01G154400
Triticum aestivum	TaPYL2D	TraesCS3D01G161500
Triticum aestivum	TaPYL3A	TraesCS7A01G358200
Triticum aestivum	TaPYL3B	TraesCS7B01G269600

Triticum aestivum	TaPYL3D	TraesCS7D01G364600
Triticum aestivum	TaPYL4A	TraesCS2A01G089400
Triticum aestivum	TaPYL4B	TraesCS2B01G105300
Triticum aestivum	TaPYL4D	TraesCS2D01G087500
Triticum aestivum	TaPYL5A	TraesCS3A01G348400
Triticum aestivum	TaPYL5B	TraesCS3B01G380300
Triticum aestivum	TaPY5D	TraesCS3D01G342000
Triticum aestivum	TaPYL6A	TraesCS1A01G297600
Triticum aestivum	TaPYL6B	TraesCS1B01G306800
Triticum aestivum	TaPYL6D	TraesCS1D01G293600
Triticum aestivum	TaPYL7A	TraesCS4A01G114400
Triticum aestivum	TaPYL7B	TraesCS4B01G189800
Triticum aestivum	TaPYL7D	TraesCS4D01G191200
Triticum aestivum	TaPYL8A	TraesCS1A01G126800
Triticum aestivum	TaPYL8B	TraesCS1B01G145800
Triticum aestivum	TaPYL8D	TraesCS1D01G126900
Triticum aestivum	TaPYL9A	TraesCS7A01G350800
Triticum aestivum	TaPYL9B	TraesCS7B01G232100
Triticum aestivum	TaPYL9D	TraesCS7D01G328000
Hordeum vulgare	HvPYL1	HORVU1Hr1G050110
Hordeum vulgare	HvPYL2	HORVU7Hr1G088140
Hordeum vulgare	HvPYL3	HORVU3Hr1G031380
Hordeum vulgare	HvPYL4	HORVU3Hr1G088100
Hordeum vulgare	HvPYL5	HORVU3Hr1G039010
Hordeum vulgare	HvPYL6	HORVU1Hr1G070420
Hordeum vulgare	HvPYL7	HORVU4Hr1G055220
Hordeum vulgare	HvPYL8	HORVU7Hr1G079270
Hordeum vulgare	HvPYL9	HORVU3Hr1G107030
Sorghum bicolor	SbPYL1	Sobic.001G289100
Sorghum bicolor	SbPYL2	Sobic.004G097800
Sorghum bicolor	SbPYL3	Sobic.010G169100

Sorghum bicolor	SbPYL4	Sobic.001G403300
Sorghum bicolor	SbPYL5	Sobic.009G170700
Sorghum bicolor	SbPYL6	Sobic.003G342000
Sorghum bicolor	SbPYL7	Sobic.009G080200
Sorghum bicolor	SbPYL8	Sobic.004G113800
Brachypodium distachyon	BdPYL1	Bradi3g34070
Brachypodium distachyon	BdPYL2	Bradi3g08580
Brachypodium distachyon	BdPYL3	Bradi1g37810
Brachypodium distachyon	BdPYL4	Bradi1g16710
Brachypodium distachyon	BdPYL5	Bradi1g65130
Brachypodium distachyon	BdPYL6	Bradi2g22510
Brachypodium distachyon	BdPYL7	Bradi2g53840
Brachypodium distachyon	BdPYL8	Bradi3g09580
Brachypodium distachyon	BdPYL9	Bradi2g32250
Ricinus comunis	RcPYRL1	XP_002519819.1
Ricinus comunis	RcPYRL2	XP_002516457.1
Ricinus comunis	RcPYRL3	XP_002526675.1
Ricinus comunis	RcPYRL4	XP_002520792.1
Ricinus comunis	RcPYRL5	XP_002523369.1
Ricinus comunis	RcPYRL6	XP_002513580.1
Ricinus comunis	RcPYRL7	XP_002509950.1
Physcomitrium patens	PpPYL9	XP_024380918.1
Physcomitrium patens	PpPYL8	XP_024366744.1
Physcomitrium patens	PpPYL9	XP_024385413.1
Theobroma cacao	TcPYRL1	XP_007039500.1
Theobroma cacao	TcPYRL2	EOY29881.1
Theobroma cacao	TcPYRL3	XP_007024469.1
Theobroma cacao	TcPYRL4	XP_007026589.1
Theobroma cacao	TcPYRL5	EOY17664.1
Theobroma cacao	TcPYRL6	XP_007025367.1
Theobroma cacao	TcPYRL7	EOY24998.1

Theobroma cacao	TcPYRL8	EOY23471.1
Theobroma cacao	TcPYRL9	XP_007051579.1
Nicotiana tabacum	NtPYL1	Ntab0128790
Nicotiana tabacum	NtPYL2	Ntab0298740
Nicotiana tabacum	NtPYL3	Ntab0331800
Nicotiana tabacum	NtPYL4	Ntab0409250
Nicotiana tabacum	NtPYL5	Ntab0524600
Nicotiana tabacum	NtPYL6	Ntab0746900
Nicotiana tabacum	NtPYL7	Ntab0790100
Nicotiana tabacum	NtPYL8	Ntab0830710
Nicotiana tabacum	NtPYL9	Ntab0986250
Nicotiana tabacum	NtPYL10	Ntab0025750
Nicotiana tabacum	NtPYL11	Ntab0143100
Nicotiana tabacum	NtPYL12	Ntab0143110
Nicotiana tabacum	NtPYL13	Ntab0350690
Nicotiana tabacum	NtPYL14	Ntab0528630
Nicotiana tabacum	NtPYL15	Ntab0012440
Nicotiana tabacum	NtPYL16	Ntab0764650
Nicotiana tabacum	NtPYL17	Ntab0430230
Nicotiana tabacum	NtPYL18	Ntab0710100
Nicotiana tabacum	NtPYL19	Ntab0177250
Nicotiana tabacum	NtPYL20	Ntab0568440
Nicotiana tabacum	NtPYL21	Ntab0424430
Nicotiana tabacum	NtPYL22	Ntab0906880
Nicotiana tabacum	NtPYL23	Ntab0217080
Nicotiana tabacum	NtPYL24	Ntab0010710
Nicotiana tabacum	NtPYL25	Ntab0725950
Nicotiana tabacum	NtPYL26	Ntab0504840
Nicotiana tabacum	NtPYL27	Ntab0868560
Nicotiana tabacum	NtPYL28	Ntab0282050

Nicotiana tabacum	NtPYL29	Ntab0734960
