# Supplementary material for: Abscisic Acid Synthesis and Signaling during the Ripening of Raspberry (Rubus idaeus ‘Heritage’) Fruit
Source: Plants (Basel). 2023 May 5;12(9):1882. doi: 10.3390/plants12091882 (PMC10180958; doi:10.3390/plants12091882)
Supplement: Supplementary file 1 [file plants-12-01882-s001.zip › Table S5.pdf]

Table S5. Information of qPCR primers

Primer's name	Sequence	Primer length (bp)	Tm	% GC	Product length (bp)
**RiNCED1-F**	TGAAAACCGTCGGGAGATA	19	59.1	47.4	154
**RiNCED1-R**	CCGTTAGGCGAGAACTTGA	19	59	52.6	
**RiPYL1-F**	AGGAGTTGAAACCCTTCGTG	20	59.2	50	159
**RiPYL1-R**	TCCGGATGAAGTGCTTGTAG	20	58.9	50	
**RiPYL8-F**	TACAAGCCCTTTGTCAGCAG	20	59.1	50	167
**RiPYL8-R**	TGATCACCACCAACAATCCT	20	58.8	45	
